# Light Accelerates Morphogenesis and Acquisition of Interlimb Stepping in Chick Embryos

**DOI:** 10.1371/journal.pone.0051348

**Published:** 2012-12-06

**Authors:** Anil Sindhurakar, Nina S. Bradley

**Affiliations:** 1 Burke-Cornell Medical Research Institute, White Plains, New York, United States of America; 2 Biokinesiology and Physical Therapy, Ostrow School of Dentistry, University of Southern California, Los Angeles, California, United States of America; Emory University, United States of America

## Abstract

Chicks are bipedal precocious vertebrates that achieve adaptive locomotor skill within hours after hatching. Development of limb movement has been extensively studied in the chicken embryo, but few studies have focused on the preparations leading to precocious locomotor skill. Chicks typically hatch after 21 days of incubation, and recent studies provided evidence that the neural circuits for intralimb control of stepping are established between embryonic days (E) 18–20. It has also been shown that variations in light exposure during embryogenesis can accelerate or delay the onset of hatching and walking by 1 to 2 days. Our earlier work revealed that despite these differences in time to hatch, chicks incubated in different light conditions achieved similar locomotor skill on the day of hatching. Results suggested to us that light exposure during incubation may have accelerated development of locomotor circuits in register with earlier hatching. Thus, in this study, embryos were incubated in 1 of 3 light conditions to determine if development of interlimb coordination at a common time point, 19 days of incubation, varied with light exposure during embryogenesis. Leg muscle activity was recorded bilaterally and burst analyses were performed for sequences of spontaneous locomotor-related activity in one or more ankle muscles to quantify the extent of interlimb coordination in ovo. We report findings indicating that the extent of interlimb coordination varied with light exposure, and left-right alternating steps were a more reliable attribute of interlimb coordination for embryos incubated in constant bright light. We provide evidence that morphological development of the leg varied with light exposure. Based on these findings, we propose that light can accelerate the development of interlimb coordination in register with earlier hatching. Our results lead us to further propose that alternating left-right stepping is the default pattern of interlimb coordination produced by locomotor circuits during embryogenesis.

## Introduction

Light exposure during embryogenesis can significantly alter the length of incubation in the chick embryo. For example, several studies observed that bright light exposure during incubation accelerated the onset of hatching by 1–2 days without altering either the rate of successful hatching or inducing morphological abnormalities [1], [2], [3], [4]. Embryos incubated in bright light exhibited weight gains of up to 17% after 4½ days of exposure, compared to embryos incubated in darkness [3]. Continuous bright light exposure during embryogenesis also appeared to accelerate development of respiratory motor control [5]. We recently found that light exposure accelerates the development of locomotion in register with the accelerated onset of hatching. Specifically, chicks that hatched early, embryonic day (E) 20, following incubation in bright light 24 hrs daily, appeared to walk with skill comparable to chicks that hatched E21–E22 following incubation in less or no light [6]. In this study, we asked if the earlier onset of locomotion was achieved in part because light exposure also accelerated development of interlimb coordination. Conversely, we considered that locomotion may be established several days before the circuitry is required for adaptive locomotion. Thus, we hypothesized that if early hatching unmasks control of interlimb stepping, reliable left-right stepping would not differ between animals at any single common time point of incubation despite differences in length of incubation. We addressed the question by examining on the 19^th^ day of incubation, the interlimb coordination for locomotion in embryos incubated in 1 of the 3 light conditions that were previously shown to significantly vary the length of incubation [6].

The characteristics of interlimb coordination during embryogenesis are a primary assay for examining locomotor circuit formation. Behavioral development of interlimb coordination has been studied in several vertebrates including the chick [7], frog [8], rat [9], [10], mouse [11] and human [12]. Surgically reduced (e.g., fictive) preparations from fetal and neonatal mouse spinal cord have been used to elucidate the neuronal connectivity for left-right and flexor-extensor control of locomotor-related leg movements [13], [14], [15]. Ventral root recordings in fictive preparations have provided a general indication of muscle contractions during locomotor behavior, one limitation being that ventral roots commonly contain axons innervating both extensor and flexor muscles [16]. Ventral root activity in fictive preparations is also somewhat artificial in that the nervous system has been stripped of the normal robust influence from both descending and afferent inputs as they begin to harness the spinal circuits for behavioral performance. These limitations underscore the continuing need for in vivo behavioral assays of locomotor development during embryogenesis. In this study, we seek to complement the results from fictive preparations by providing direct evidence of emerging interlimb coordination in the intact and spontaneously active chick embryo prior to the onset of locomotion at hatching.

In the chick, evidence suggests that at least some components of the neural circuitry for locomotion are established prior to hatching. Chick embryos begin to move partially formed limbs by E4, and by E7, they can flex and extend the leg [17], [18]. By E9, the leg movements are accompanied by alternating flexor and extensor muscle activity and coupled joint rotations at the hip, knee and ankle [19], [20], [21]. Recent EMG and kinematic studies in chicks report a distinct leg motor behavior called repetitive limb movement (RLM) that emerges between E15–E18 [22], [23]. RLMs have a frequency range of 1–10 Hz that is comparable to EMG and kinematic data for leg movements during airstepping, walking and swimming in hatchlings [24]. Given that chicks are precocious walkers, locomotor circuits for interlimb stepping are presumably assembled during embryogenesis. Limited evidence suggests that embryos preparing to hatch at E21 initiate alternating (left-right) steps at E20, 1 day prior to hatching [23]; however the emergence of interlimb coordination during embryogenesis remains to be fully understood. Thus, one goal of this study was to experimentally determine if alternating interlimb coordination for locomotion in chick is also a reliable feature of stepping at least 1 day before hatching at E20.

In this study, we provide the first experimental examination of bilateral stepping during embryogenesis in the chick and evidence that light exposure can significantly impact its development. Our results also extend reports that light exposure during embryogenesis can broadly impact morphological and physiological development. We discuss our findings within a developmental framework that seeks to account for precocious overground locomotion at hatching. We propose that there is an orderly sequence of motor control development leading to precocious walking at hatching in which intralimb stepping precedes interlimb stepping, and precise alternating steps of the left and right legs gradually emerge. We also extend earlier observations and discussions of flexor bias in leg EMG activity just before hatching.

## Methods

### Subjects

Fertile Leghorn chicken (*Gallus gallus*) eggs were obtained from a local hatchery and incubated in poultry incubators. The incubators were maintained at standard temperature (37.5°C) and humidity (62%), and the egg racks rotated automatically at 2 hour intervals, according to manufacture guidelines (GQF Manufacturing, Savannah, GA). Onset of incubation was considered embryonic day E0. Data were collected on E19. Embryos were maintained in a humidified, heated chamber during preparation and recording, and euthanized at the end of data collection. Left and right tibias were collected and fixed in 10% formalin for measurement of bone length as an estimate of physical size on the day of experimentation.

### Ethics Statement

All care and experimental procedures were approved by the University Institutional Animal Care and Use Committee for University of Southern California in strict accordance with the Guide for the Care and Use of Laboratory Animals of the National Institutes of Health.

### Incubation Conditions

Fertilized eggs were weighed and randomly assigned to 1 of 3 light exposure conditions during incubation from E0 to E19. The 3 conditions replicated those established for an earlier study [6]: continuous light exposure 24 hrs daily (24L) at 4000–7000 lux; 12 hrs light exposure daily (12L) at 650–3000 lux; or continuous dark exposure 24 hrs daily (24D) at ≤1 lux. Lux ranges were based on 27 measures per condition as follows. Illuminance was measured at the approximate height of the egg at 9 locations on each incubator rack (left, right and midpoint at the front, center and back of the incubator) while the rack was in each of 3 rotation positions (tipped left, right and horizontal). Standard temperature and humidity were maintained under all conditions, though we cannot discount possible differences in egg internal temperature. Collectively, the 3 light conditions were based on published evidence that they significantly varied the time to hatching and onset of locomotion [2], [3], [6], [25], [26]. In addition, the 12L condition served as an experimental control, for the incubators were located within our primary lab space, wherein light exposure varies throughout the day and week with lab activities. The 12L condition stabilized routine light exposure, and reliably incubated embryos that hatched at E21, typical for chicken embryos [27]. Thus, the 12L conditions were viewed as a reasonable laboratory reference for comparisons across incubation conditions that reliably varied the time to hatch.

### EMG and Video Recordings

To prepare the embryo for EMG implantation, a window was created by removing a small region of the shell and dissecting the membranes to expose the posterior surface of the left and right lower legs. Two muscles, an ankle extensor (lateral gastrocnemius, LG) and an ankle flexor (tibialis anterior, TA) were implanted bilaterally with bipolar electrodes fabricated from silver fine wire (o.d. 50 µm). A minutin pin was also inserted into the posterior surface of each ankle so that ankle displacements could be later digitized for select EMG sequences in data presentations. However, these data could not be corrected for movement out of plane and therefore were not suitable for quantitative kinematic analyses.

After implanting the ankle muscles, the embryo was transferred to a Styrofoam® recording chamber on an anti-vibration table. A video camera was placed directly above the chamber lid to record limb movements. EMG and video recordings were continuous for the duration of the experiment. EMG and video data were stored directly to disk in 10 min increments (Datapac 2K2, Run Technologies). A synchronizing pulse was manually triggered every 5 min and the output was directed to both the EMG and video recordings for off-line data synchronization. EMG signals were bandpass filtered (100–1 kHz), amplified (X2000), and computer sampled at 4 kHz (16, 17). The accuracy of EMG implant locations were verified by dissection immediately after euthanizing the embryo.

### EMG Analyses

**Figure 1:**
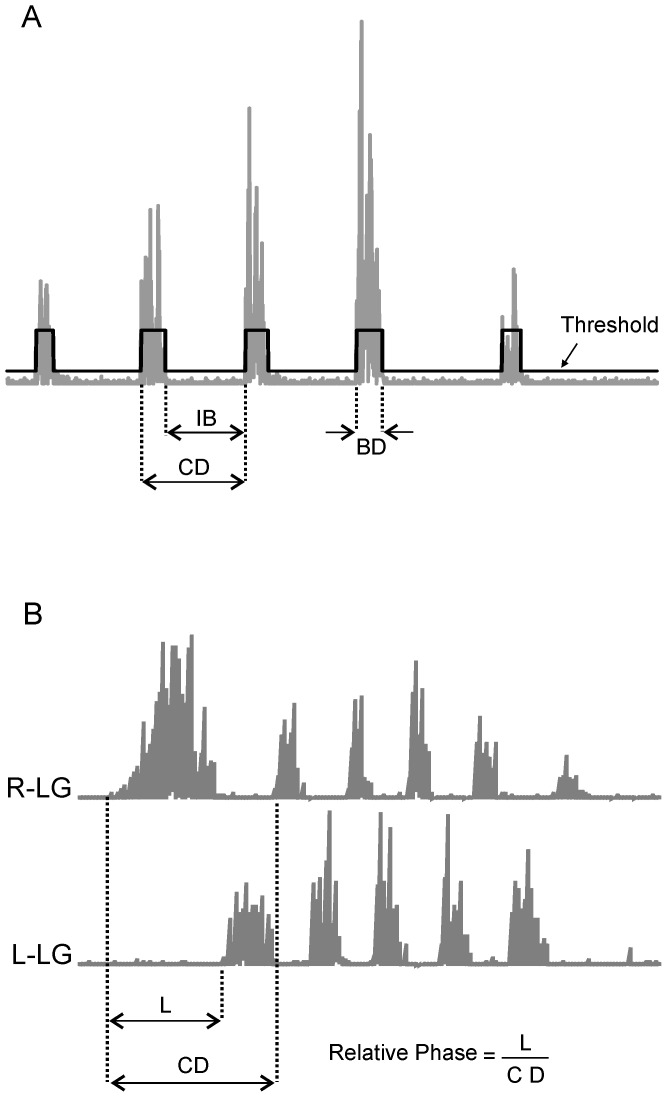
Burst detection parameters and relative phase calculations for EMG signals. Automated burst detection identified all possible events in each rectified EMG trace that met pre-defined criteria characteristic of repetitive limb movements (RLMs). A: Burst detection employed 4 parameters: burst duration (BD), inter-burst interval (IB), cycle duration (CD) and threshold. In this sequence, a threshold of 20 µV, burst duration of 20–1000 ms, and an inter-burst interval ≥20 ms detected 5 TA bursts and 4 TA cycles. Parameter definitions are provided in the text. B: Methods for calculating relative phase for concurrent RLM sequences in the right LG (R-LG) and left (L-LG) are illustrated. In this example, the R-LG RLM was selected as reference for bilateral RLM analyses. L-LG burst onset latency (L) was measured from onset of the preceding R-LG cycle, and L-LG latency was divided by R-LG CD to obtain L-LG relative phase. The RLM (left or right) selected as reference for relative phase calculations varied across consecutive bilateral RLMs as explained in the text.

All spontaneous, repetitive limb movement sequences (RLMs) per experiment were located from documentation during data collection and off-line review of all associated EMG files. EMG signals were rectified for automated burst detection (Datapac 2K2, Run Technologies). Burst detection employed 3 parameters established in earlier studies [22], [23]: burst threshold, burst duration and inter-burst interval (Fig. 1A). Burst threshold was defined as EMG activity 2–4 times greater than baseline noise; the threshold that most accurately captured RLM bursts was selected for all analyses of that channel. Burst duration was defined as EMG activity remaining above threshold for 20–1000 ms. Inter-burst interval was defined as activity remaining below threshold 20 ms or longer. Together the 3 detection parameters were used to identify cycle duration, the time between onsets of 2 consecutive bursts (Fig. 1A).

Analyses were restricted to TA and/or LG EMG sequences of bursting at 1–10 Hz, the range identified as locomotor-related RLMs in our previous studies [22], [23]. When a higher burst frequency was identified within a sequence that was otherwise in the RLM range, the second burst was treated as a double burst within the longer cycle. RLMs containing 4 or more consecutive bursts in at least one muscle were retained for interlimb analyses. The consecutive burst onsets in one flexor or extensor (e.g., reference muscle) were used to quantify the relative timing of burst onsets in the homologous contralateral muscle (Fig. 1B). However, as reported previously [22], [23], the combination of muscles repetitively active varied from sequence to sequence, so the muscle having the greatest number of consecutive bursts was selected as the reference for that bilateral RLM sequence as a strategy to maximize the within-animal sample size. Burst onset latency in the contralateral muscle was then subtracted from the preceding burst onset of the reference muscle and divided by the concurrent cycle duration in the reference muscle to obtain the relative phase (Fig. 1B). Relative phase values of 0 and 1 were indicative of synchronous bilateral bursts and 0.5 was indicative of symmetrically alternating bursts.

### Statistical Analysis

Tibia length was compared across incubation groups using the two-way Analysis of Variance and the t-test Two-Sample for Means Assuming Equal Variance for post hoc analyses, as these data sets appeared to be normally distributed. However, data sets for EMG burst analyses were non-normally distributed, thus nonparametric tests were used for comparisons of all burst parameters [28]. The Kruskal-Wallis one-way Analysis of Variance was used for between-group comparisons and the Mann-Whitney U-test one-way Analysis of Variance was used for within-group comparisons (SPSS Inc., Chicago, IL). Circular statistics were used to determine the phase relationship between reference TA or LG bursts and bursts in the contralateral homologous muscle, and to compare phase data across incubation groups [29]. The Rayleigh’s test was used to determine if the relative phase for contralateral burst onsets was significantly different from a random distribution. The Watson-Williams test was used to compare the distribution in relative phase across groups, and the Mann-Whitney U test was used for post-hoc analyses. Statistical significance was set at p<0.05 and a Bonferroni correction was applied for multiple comparisons.

## Results

Results summarize findings at E19 for a total of 10 embryos per incubation condition (24L, 12L or 24D). We first report findings indicating that morphologic development of the leg differed across the 3 conditions. We then summarize findings for analyses of 904 RLM sequences indicating that interlimb coordination of leg stepping also differed across incubation conditions.

### Tibia Bone Length Varied with Light Exposure

**Figure 2:**
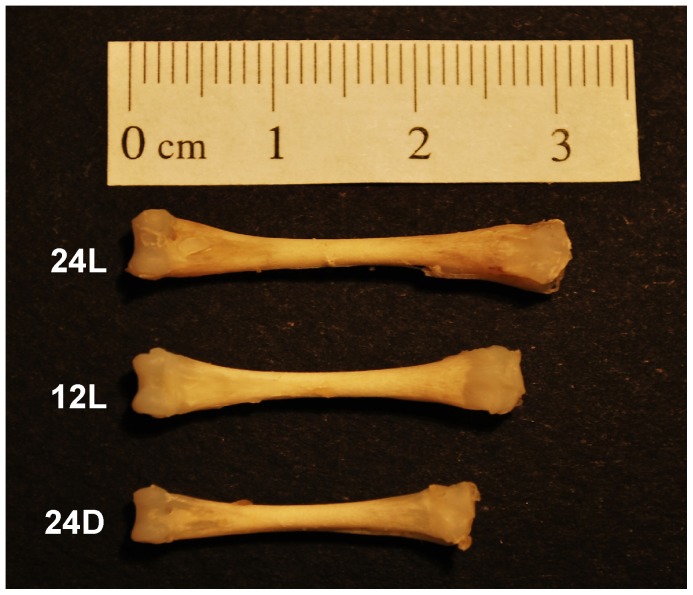
Tibia length at E19. Both left and right tibias were fixed in formalin and the lengths later measured. The 3 tibias shown illustrate the significant differences across incubation conditions for bone length (F_2,54_ = 133.3, p<0.001). Tibia length was greatest for 24L incubation (top) and least for 24D incubation (bottom). See text for further details.

Tibia bone length varied with light exposure during embryogenesis (Fig. 2). Tibia length was greatest for embryos incubated in 24L and least for embryos in 24D. A two-way ANOVA indicated that the differences in left and right tibia lengths across conditions were significant (F_2,54_ = 133.3, p<0.001) but length did not vary between limbs (F_1,54_ = 0.003, p>0.95). Post hoc t test comparisons for left tibia indicated that length was greater after 24L incubation than 12L (p<0.001) and greater after 12L incubation than 24D (p<0.001). (See Table S1 in supplementary material for a detailed summary.).

### RLM Classification

**Figure 3:**
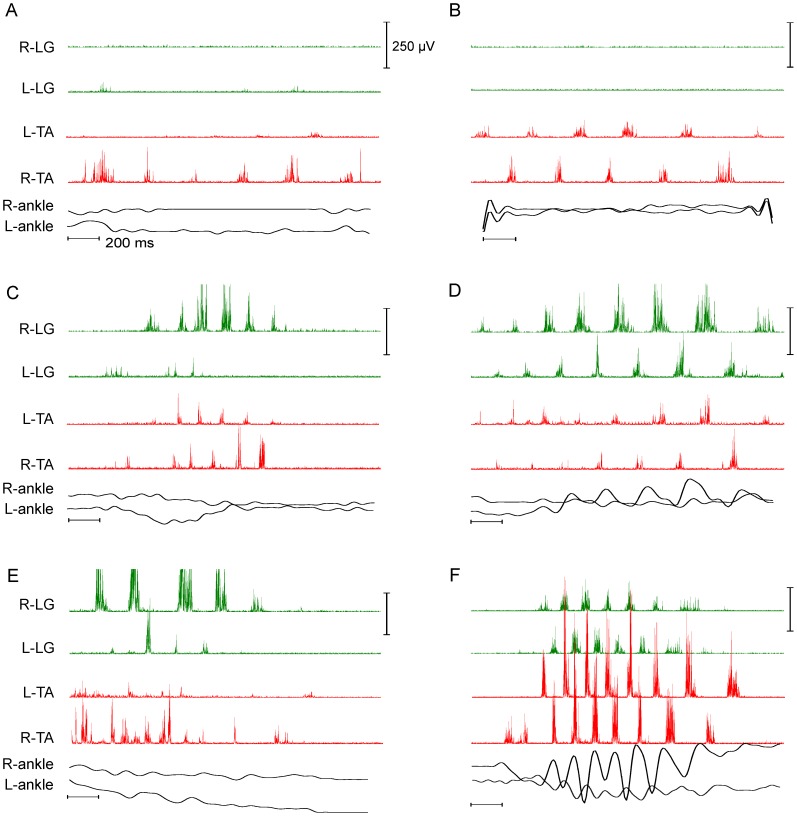
Variations in RLM EMG patterns at E19. Samples of RLMs from a single 24L experiment illustrate the combinations of muscle activity in EMG recordings commonly observed. A: The 6-burst sequence in R-TA RLM was unaccompanied by EMG activity in other muscles. B: The 6-burst RLM in R-TA was accompanied by a 6-burst sequence in L-TA. C: RLM sequences were generated concurrently in 3 muscles: R-LG, L-TA and R-TA. D: An 8-burst RLM in R-LG was accompanied by a 7-burst RLM in L-LG, and 4-burst RLMs in both L-TA and R-TA. Onset of L-LG bursts alternated with onset of R-LG bursts. E: The R-LG RLM was accompanied by extensive non-RLM activity in R-TA. F: Well-formed RLMs were expressed concurrently in all 4 EMG traces, and alternating left-right burst onsets were distinct in LG RLMs and TA RLMs. Also, L-LG and L-TA burst onsets alternated. RLM burst frequency also varied across RLMs within experiments, e.g., burst frequencies in D were slower than frequencies in F. Scales A–F: Vertical scale bars equal 250 µV (EMG) and 0.06 mm (ankle displacement). Horizontal scale bars equal 200 ms.

Spontaneous RLM muscle activity varied over the course of all experiments, regardless of incubation condition, as illustrated by 6 RLMs for one experiment in Fig. 3. For example, an RLM sequence occurred in one TA while the contralateral TA and both LGs remained mostly silent (Fig. 3A). In another RLM, both TAs produced RLM sequences and both LGs remained silent (Fig. 3B). In yet other RLMs, bilateral TA RLM sequences were accompanied by RLM bursts in the left LG (Fig. 3C) or both LGs (Fig. 3D, 3F). During other RLMs, an RLM sequence in one muscle was accompanied by 1–3 RLM-like bursts or a single tonic burst in other muscles (Fig. 3E); or the tonic burst preceded bilateral TA and LG sequences (Fig. 3F). Owing to the diversity in RLM burst patterns, we sorted all RLM sequences per experiment into 1 or 2 classes: bilateral TA (Fig. 3B–3D, 3F), bilateral LG (Fig. 3D, 3F), unilateral TA (Fig. 3A) or unilateral LG (Fig. 3C, 3E).

### Incidence of Bilateral and Unilateral RLMs Varied with Light Exposure

**Figure 4:**
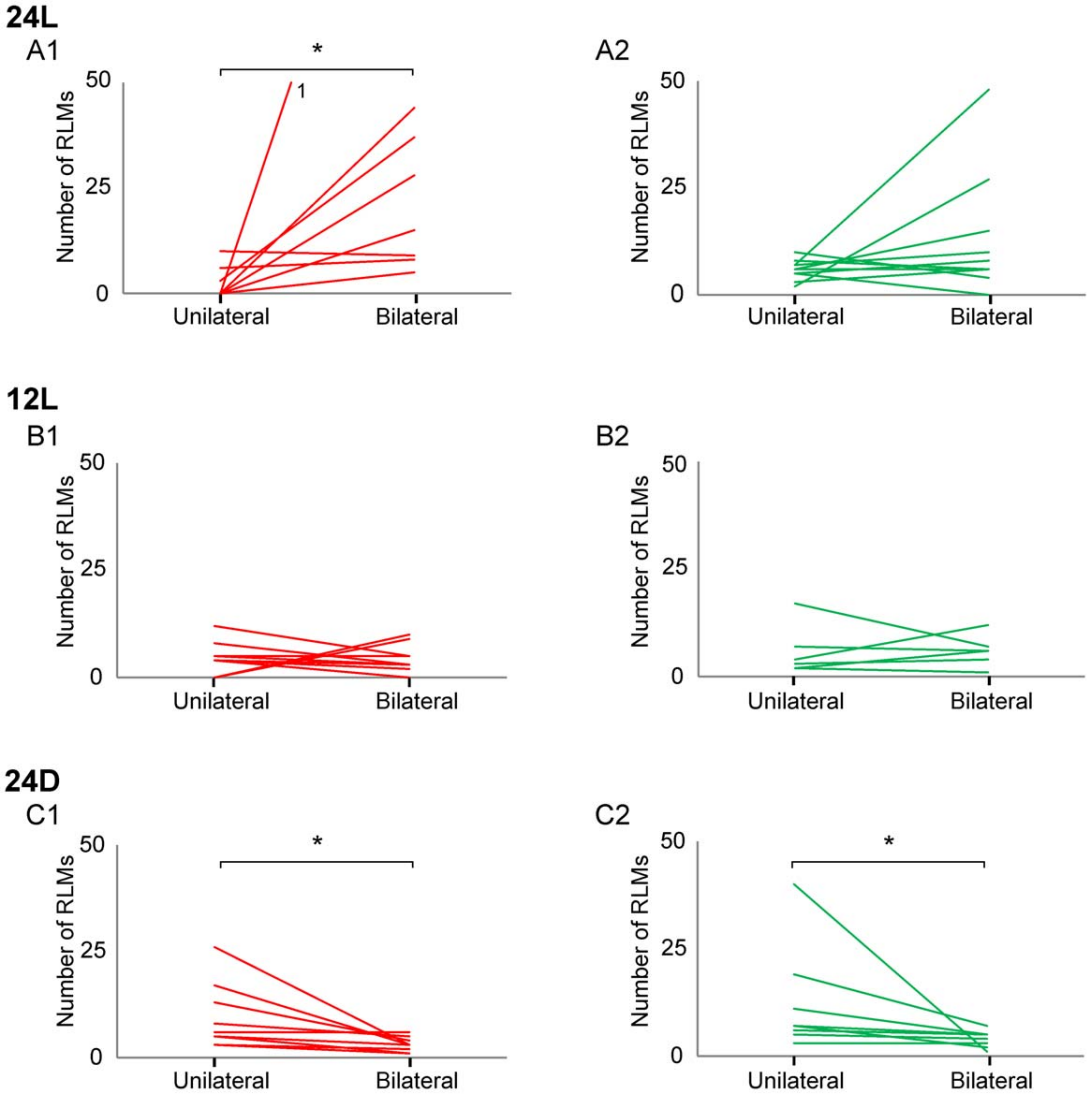
Incidence of unilateral and bilateral RLM bursts. Line plots identify the total number of unilateral and bilateral RLMs produced per experiment for TA (red plots to left) and LG (green plots to right). The plots illustrate the differences in distribution of unilateral and bilateral RLMs observed across incubation conditions: 24L (A1, A2), 12L (B1, B2) and 24D (C1, C2). Note that recording time varied across experiments; data normalized by experiment duration are provided in Table 2. Asterisks identify significant within-subject differences for incidence of unilateral and bilateral RLMs. A1: U = 4.0, p<0.003; C1: U = 13.5, p<0.01; C2: U = 10.5, p<0.01. A1: The superscript (^1^) identifies an experiment in which the total number of bilateral TA RLMs (137 RLMs) greatly exceeded an appropriate scale for all other data.

Both the distribution of bilateral and unilateral RLMs and the relative incidence of RLMs during experiments varied with light exposure during embryogenesis. Bilateral RLMs were more commonly observed in 24L experiments than all other experiments (Fig. 4). Both the number of bilateral TA and LG RLMs exceeded unilateral TA or LG RLMs (Fig. 4A1, 4A2). Within-group analyses for 24L experiments indicated that the biased distribution of bilateral versus unilateral TA RLMs was significant (U = 4.0, p<0.003, n = 8). A similar bias for bilateral LG RLMs was observed in 6 experiments, but the within-group comparison was non-significant (U = 35.0, p>0.3, n = 10). To test for differences in the distribution of RLMs across groups, we normalized RLM counts by experiment duration (Table 1). Comparisons indicated that the incidence of bilateral RLMs significantly varied across groups for TA sequences (χ^2^ = 10.5, p<0.006) but not for LG (χ^2^ = 1.6, p>0.4). The Mann-Whitney post-hoc comparison (Bonferroni correction, p<0.017) indicated that embryos incubated in 24L produced significantly more bilateral TA RLMs than embryos in 24D (p<0.003); the difference between embryos in 24L and 12L fell short of significant (p<0.021).

**Table 1:** Bilateral and unilateral RLMs normalized to recording time.

Incubation Condition	Bilateral TA RLMs/hr^1^	Unilateral TA RLMs/hr^2^	Bilateral LG RLMs/hr	Unilateral LG RLMs/hr
**24L**	7.5±7.8	0.5±0.7	2.6±2.7	1.1±0.4
**12L**	1.5±1.1	1.5±1.4	1.5±1.3	1.4±1.1
**24D**	0.9±0.5	2.7±1.8	1.1±0.8	2.7±1.4

1 Kruskal-Wallis, main effect for incubation condition, p<0.006.

2 Kruskal-Wallis, main effect for incubation condition, p<0.02.

In contrast to embryos incubated in 24L conditions, embryos incubated in 24D produced more unilateral than bilateral TA RLMs (Fig. 4C1) and LG RLMs (Fig. 4C2). Mann-Whitney U tests indicated these biased distributions were significant for both TA (U = 13.5, p<0.01, n = 9) and LG RLMs (U = 10.5, p<0.01, n = 8). After normalizing by length of recording (Table 1), the Kruskal-Wallis test indicated that the distribution in unilateral RLMs significantly varied across incubation conditions for unilateral TA RLMs (χ^2^ = 9.0, p<0.02), but not LG RLMs (χ^2^ = 4.6, p>0.1). Post-hoc comparisons (Bonferroni correction, p<0.017) indicated that embryos incubated in 24D produced more unilateral TA RLMs than embryos incubated in 24L (U = 8.0, p<0.007); the difference between embryos in 24D and 12L did not achieve significance (p<0.054).

Embryos exposed to intermediate levels of light during embryogenesis (12L) produced similar numbers of unilateral and bilateral TA (Fig. 4B1) and LG RLMs (Fig. 4B2). Mann-Whitney comparisons indicated there were no differences in incidence of unilateral and bilateral TA (U = 43, p>0.6) or LG RLMs (U = 23.0, p>0.9).

Given the differences in distribution of bilateral and unilateral RLMs and variations in TA and LG participation, we asked if RLM burst frequencies also varied with these distributions. Results indicated frequencies were similar for bilateral and unilateral TA and LG RLMs across conditions (Table 2). For example, burst frequency ranges for TA RLMs was 2–8 Hz (unilateral RLMs) and 3–7 Hz (bilateral RLMs). Unilateral and bilateral LG RLMs exhibited similar burst frequency ranges (Table 2), and results for Kruskal-Wallis comparisons indicated there were no differences in burst frequency across incubation conditions for either bilateral or unilateral TA or LG RLMs.

**Table 2:** Burst frequency distribution for unilateral and bilateral RLMs.

	24L	12L	24D
**TA Burst Frequency (Hz)**			
Unilateral R-RLM	4.9±3.2	5.1±2.2	4.6±1.1
Unilateral L-RLM	4.6±2.6	4.9±0.8	4.0±2.3
Bilateral RLM^1^	4.6±1.3	3.9±0.7	3.7±0.7
**LG Burst Frequency (Hz)**			
Unilateral R-RLM	5.3±1.7	4.8±0.3	4.6±1.7
Unilateral L-RLM	6.2±1.5	4.9±1.8	4.9±1.3
Bilateral RLM^1^	5.5±1.2	3.9±0.7	3.9±1.8

Values represent mean ± SD.

1 Burst frequencies of the reference leg during phase analysis.

### Relative Phase of Bilateral RLM Activity Varied with Light Exposure

**Figure 5:**
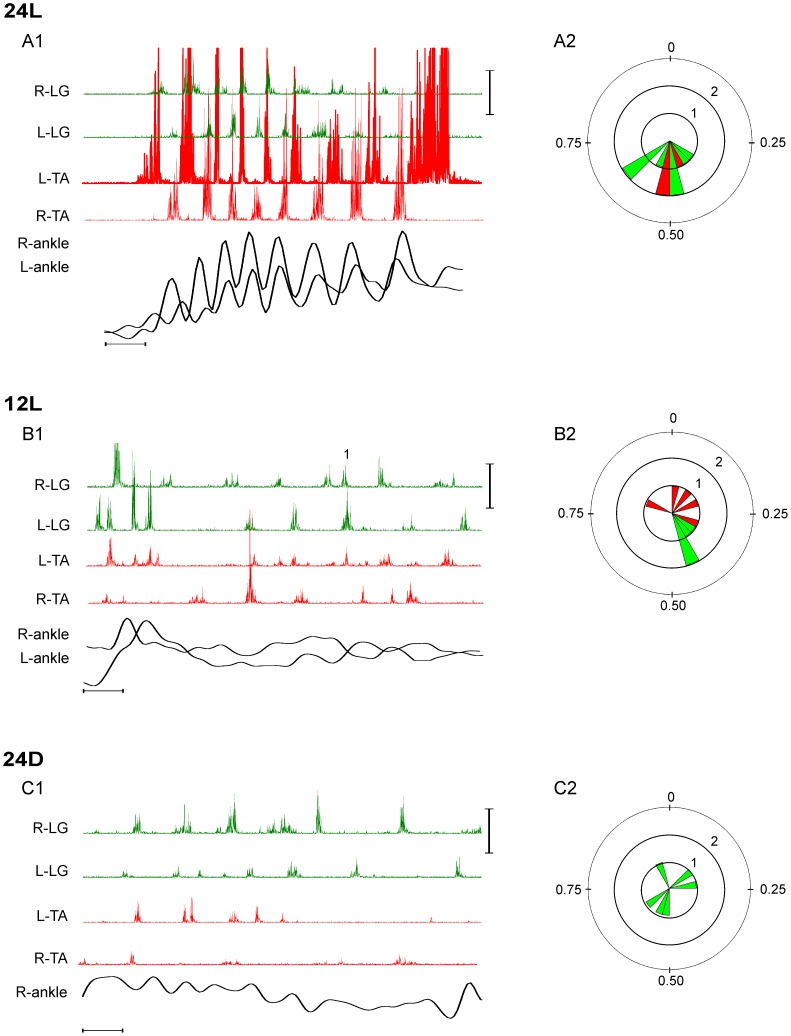
Rose plot representation of relative phase analyses. The rose plots summarize, in a circular histogram format, the relative phase analyses for contralateral TA (red petals) and LG burst onsets (green petals). Exemplary bilateral RLMs and corresponding rose plots are shown for each of the 3 incubation conditions: 24L (A), 12L (B) and 24D (C). Petals pointing to 0 indicate that the TA or LG burst in the contralateral RLM and homologous burst in the reference RLM began synchronously. Petals pointing to 0.5 indicate that burst onset in the contralateral TA or LG RLM and homologous burst in the reference RLM symmetrically alternated. The length of the petal, scaled by concentric rings, indicates the number of bursts observed for a given phase. In the examples shown, the rings indicate a scale of 1–3 contralateral bursts per petal. A: The relative phases for 7 R-TA and 7 R-LG bursts in A1 are plotted in A2; petals for 4 TA bursts are hidden by LG petals: 2 TA bursts at 0.46, 1 burst at 0.41 and 1 at 0.35. The average relative phases for R-TA and R-LG bursts were 0.45, and 0.50, respectively. B: The relative phases for 5 L-TA bursts and 4 L-LG bursts in B1 are plotted in B2. The average relative phases for L-TA and L-LG were 0.28 and 0.40, respectively. The superscript (^1^) in B1 identifies a double burst (burst frequency >10 Hz) in the 3^rd^ R-LG cycle (see Methods for further analysis details). C: The relative phases for 6 R-LG in C1 are plotted in C2. The average relative phase was 0.51. Scaling: vertical bars equal 250 µV (EMG amplitude) and 0.06 mm (ankle displacement). Horizontal bars equal 200 ms.

Sequences of bilateral RLM activity readily distinguished EMG recordings for embryos incubated in 24L conditions from all other recordings. This was primarily because bilateral RLMs were characterized by distinctly alternating bursts in the left and right TA and LG EMG. In Fig. 5A1 for example, the left TA (L-TA) was the reference muscle, and generated 8 RLM bursts forming 7 TA cycles; 7 of 8 RLM bursts in the right TA (R-TA) fell within these cycles. Each R-TA bursts began near the midpoint of the concurrent L-TA cycle. Similar trends were detected in the left and right LG (L-LG, R-LG) RLMs. The relative phase for each R-TA and R-LG burst is included in the petals of the adjacent rose plot (Fig. 5A2), though some LG petals (green) overlay TA petals (red). The average relative phase for the R-TA bursts was 0.45±0.06 and the average for R-LG bursts was 0.50±0.11. In contrast, the relative phase for bilateral RLMs was rarely alternating in EMG recordings for embryos incubated in 12L or 24D conditions. Typically, in these latter experiments, the relative phase was broadly distributed for burst onsets in the contralateral TA and LG, as exemplified by the R-TA EMG for an embryo incubated in 12L (Fig. 5B1, 5B2) and the L-LG for an embryo incubated in 24D (Fig. 5C1, 5C2).

**Figure 6:**
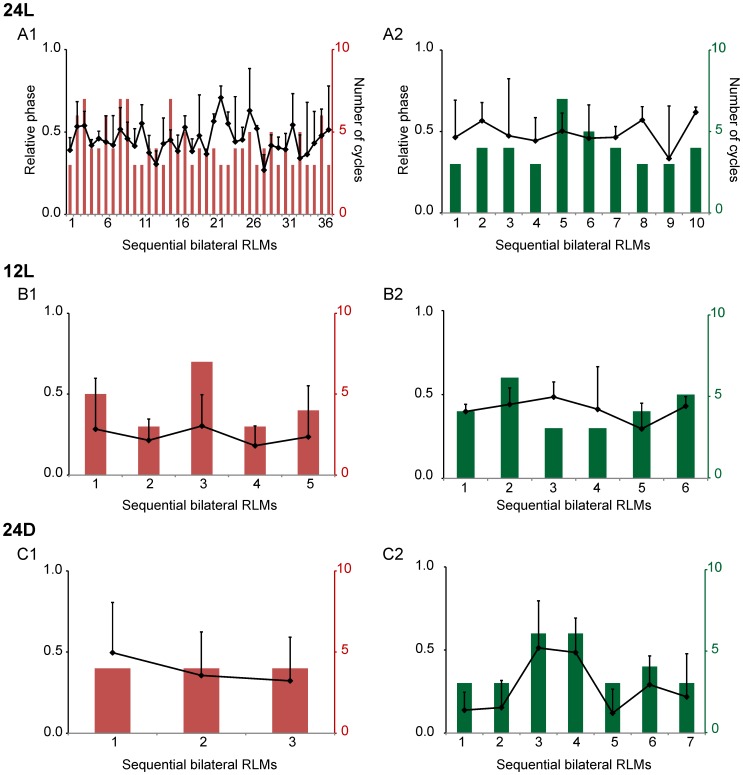
Variation in average relative phase across sequential bilateral RLMs. Plots of bilateral RLM relative phase analyses, serially ordered, illustrate the typical variations observed within experiments for TA (left) and LG (right) burst sequences. Each TA and LG pair represents one experiment per condition: 24L (A), 12L (B) and 24D (C). Black markers and lines indicate the average relative phase plus standard deviation (SD), referenced to the left axis in black. Red and green columns indicate the number of TA and LG cycles per RLM, respectively, referenced to the right axis (red or green). The 3 RLMs examined in Fig. 5A–C were drawn from these 3 experiments.

Experiments were several hours in length, so we examined relative phase across sequential RLMs for possible trends within experiments, as well as between experiments. In Fig. 6, to illustrate, sequential bilateral TA and LG RLMs are plotted for 3 experiments, one per incubation condition (A–C). In each experiment, the contralateral RLM typically consisted of 3 to 5 TA (red bars) or LG cycles (green bars); sequences of 6 or more cycles were more common for 24L conditions (Fig. 6A). The average relative phase was calculated for each RLM (black markers connected by a black line). An average relative phase between 0.4–0.6 was considered approximately symmetric interlimb alternation. In the 24L experiment (Fig. 6A), the average relative phase per RLM ranged from approximately 0.3 to 0.7, and fell between 0.4–0.6 for 67% of the TA RLMs (Fig. 6A1) and 80% of the LG RLMs (Fig. 6A2). Far fewer bilateral RLMs occurred during the 12L (Fig. 6B) and 24D experiments (Fig. 6C), which was typical for these groups (Table 2). In the 24D experiment shown, only 33% of contralateral TA RLMs (Fig. 6C1) and 28% of LG RLMs (Fig. 6C2) yielded averages between 0.4–0.6. In the 12L experiment, no TA RLMs fell within the 0.4–0.6 range (Fig. 6B1), but >80% of LG RLMs fell within range (Fig. 6B2).

**Figure 7:**
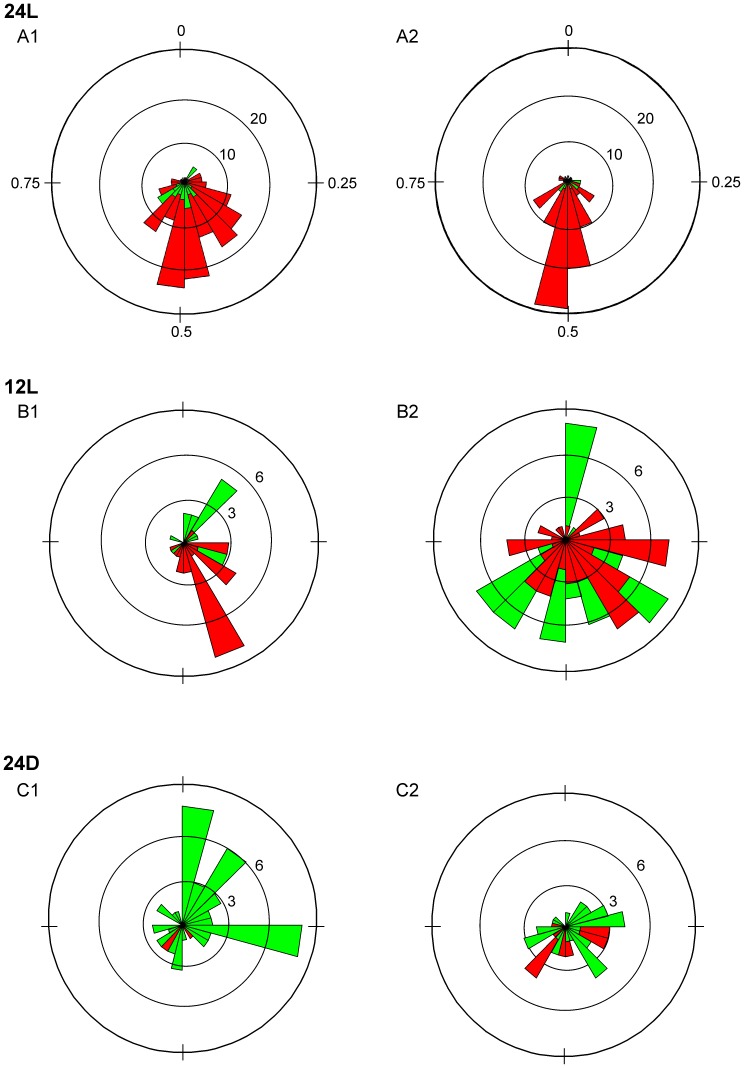
Rose plot summaries for distributions in TA and LG relative phase during bilateral RLMs. Individual rose plots summarize within experiment the relative phase distributions for both TA RLMs (red petals) and LG RLMs (green petals). Results for 2 experiments per incubation condition are shown: 24L (A), 12L (B) and 24D (C). In general, sample sizes were greatest for TA RLMs. TA petals were concentrated within the range of 0.4–0.6 for A1 and A2. Note that scales for concentric rings vary to account for the larger sample sizes in experiments for 24L conditions: 0–30 contralateral bursts (24L) and 0–9 bursts (12L and 24D). Sample sizes for each plot were as follows for TA and LG bursts, respectively: A1 (157, 40), A2 (108, 21), B1 (22, 25), B2 (66, 58), C1 (12, 51), and C2 (22, 32). Average relative phase and SD for TA and LG, respectively were: A1 (0.46±0.09, 0.49±0.08), A2 (0.50±0.05, 0.43±0.05), B1 (0.26±0.22, 0.41±012), B2 (0.45±0.22, 0.47±0.21), C1 (0.37 0.29, 0.31±0.27), and C2 (0.46±0.17, 0.39±0.23).

**Figure 8:**
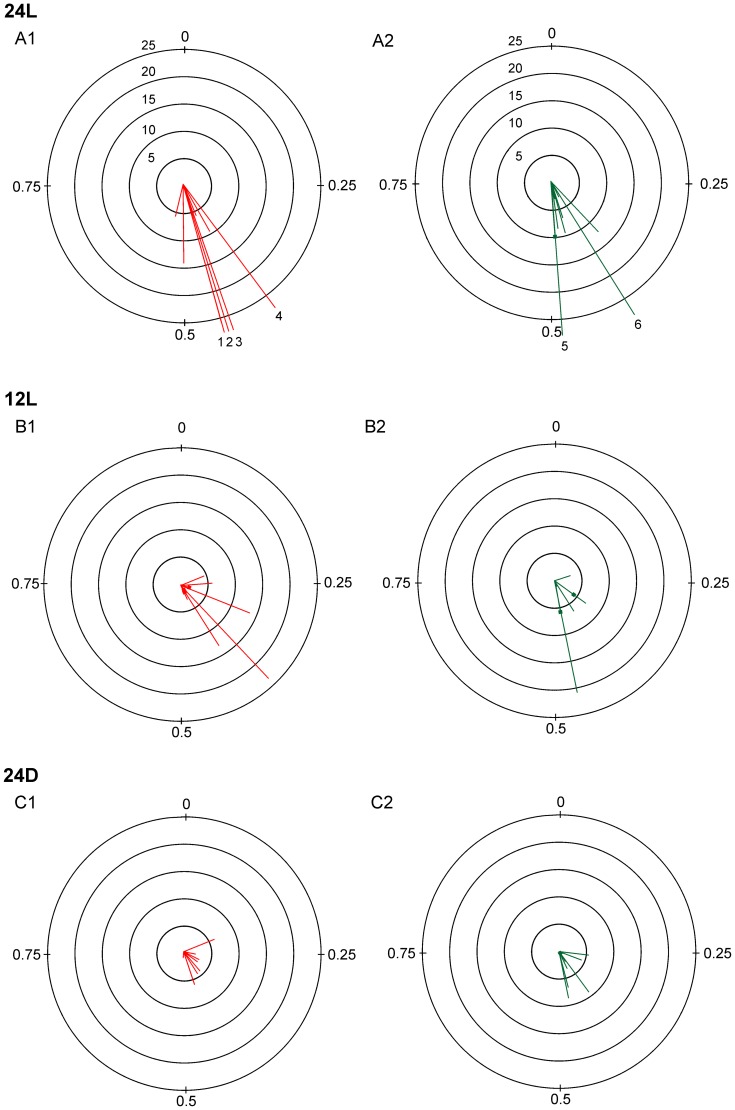
Average relative phase plots for bilateral RLMs. Circular plots summarize the results of relative phase analyses for all experiments. Each vector represents either the TA (red) or LG (green) RLMs for 1 experiment. Results are plotted for all experiments per incubation condition: 24L (A), 12L (B) and 24D (C). Vector direction represents the average relative phase. Vector length represents the relative incidence of bilateral TA or LG cycles, and is expressed as the average number of reference cycles/hr (the scale of 0–25 bursts applies to all plots). In several experiments for 24L conditions (A), the number of bilateral RLM cycles greatly exceeded the ranges for most experiments; they are identified by numbered vectors that extend beyond the outer ring. The adjusted sample sizes for the numbered vectors were as follows: 1 (40), 2 (124), 3 (29), 4(90), 5(43) and 6(40). Vectors distinguished by a small square symbol indicate the length of a second, shorter vector overlying a longer vector for the same direction. bilateral TA and LG RLMs across the 3 incubation conditions.

We found no trends in relative phase over the course of an experiment, as illustrated by the 3 experiments in Fig. 6. Thus, we pooled within embryo the relative phase of all TA or LG burst onsets in the contralateral RLM to obtain the average TA and LG relative phase for comparisons between incubation conditions. The distribution in relative phase of individual cycles and number of cycles produced suggested that embryos incubated in 24L more frequently produced bilateral RLMs that were symmetrically alternating. For example, in Fig. 7 all TA burst onsets (red) and LG burst onsets (green) for the 3 experiments in Fig. 6 are represented in rose plots on the left (Fig. 7A1, 7B1, 7C1); to the right are plots for 3 additional experiments per condition (Fig. 7A2, 7B2, 7C2). In the 2 experiments for 24L conditions (Fig. 7A1, 7A2), the relative phase fell between 0.4–0.6 in 30–71% of TA and LG cycles. In contrast, the relative phase for TA and LG burst onsets was more broadly distributed for the 2 embryos incubated in 12L (Fig. 7B1, 7B2) and 24D conditions (Fig. 7C1, 7C2). In Fig. 7B1 and 7B2, relative phases fell between 0.4–0.6 in 0–56% of TA and LG cycles; and in Fig. 7C1 and 7C2, only 11–41% fell within this range. In addition, a far greater number of cycles where generated during bilateral RLMs in both 24L experiments (7A1, 7A2), as compared to those for 12L and 24D experiments, and is indicated by the greater scale range for concentric rings in Fig. 7A, compared to Fig. 7B and 7C. The average relative phase and number of cycles produced in the contralateral TA or LG during bilateral RLMs is plotted for all experiments in Fig. 8. In 24L conditions, the average relative phase fell between 0.4–0.6 in 8 of 8 experiments for bilateral TA RLMs and 8 of 9 experiments for LG (Fig. 8A1, 8A2, Table S2). In 12L conditions, average relative phase fell between 0.4–0.6 in only 4 of 9 experiments for TA and 4 of 7 experiments for LG RLMs (Fig. 8B1, 8B2). In 24D conditions, average relative phase fell midrange for TA RLMs in only 3 of 9 experiments and for LG RLMs in 5 of 8 experiments (Fig. 8C1, 8C2). See Table S4 for further vector details.

Circular statistics were used to compare across conditions the distributions in relative phase of TA and LG bursts during bilateral RLMs, and these results also suggested that there were some differences in the distributions. The Rayleigh’s test indicated that for 24L conditions, the distribution in relative phase was significantly different from random for bilateral TA RLMs in 8 of 8 experiments and LG RLMs in 9 of 9 experiments (Tables S2, S3). In contrast, for 12L conditions, the distribution in relative phase for bilateral TA RLMs was significantly different from random in 5 of 9 experiments and for LG in 4 of 6 experiments. Finally, for 24D conditions, the distribution for TA relative phase differed from random in only 4 of 7experiments and for LG in only 1 of 7 experiments (Table S3). Regardless of these differences in distribution, Watson-William tests indicated that mean relative phase did not differ across groups for either bilateral TA RLMs (F = 2.55, p>0.09) or LG RLMs (F = 2.24, p>0.1). Further, we observed that the distributions in relative phase for experiments with fewer than 40 bilateral cycles were less likely to differ from a random distribution (Table S3) and cycle numbers were generally lower for both 12L and 24D conditions. Comparisons confirmed that there was a significant difference across conditions for the number of bilateral TA cycles (χ^2^ = 14.7, p<0.001) and LG cycles (χ^2^ = 3.8, p<0.02). Post-hoc comparisons (Bonferroni correction, p<0.017) revealed that experiments for both 12L (p<0.004) and 24D (p<0.001) produced fewer bilateral TA cycles than experiments for 24L. Also, experiments for 24D (p<0.012) produced fewer bilateral LG cycles than experiments for 24L condition, but the comparison for 12L and 24L fell short of significant (p>0.06). Thus, differences in distribution of relative phase may have been due to differences in sample size.

## Discussion

In this study we provide the first experimental examination of interlimb coordination in the intact chick embryo during spontaneous activity 1–3 days prior to hatching and onset of bipedal locomotion. Our results indicate that the embryo incubated in bright light 24 hrs daily (24L) can produce reliable sequences of alternating left-right stepping at least 1 day prior to hatching. Our results for embryos incubated in less (12L) or no light (24D) indicate that interlimb coordination is also present, but to a lesser extent, at least 2–3 days before hatching and that unilateral stepping is more reliably expressed than bilateral stepping at this time point. Our findings also provide the first evidence that light exposure during embryogenesis may accelerate the development of neural circuits for control of interlimb coordination. We consider how these findings contribute to extending our knowledge of locomotor control development.

### Light Exposure during Embryogenesis Accelerates Development

The differences in tibia bone length observed across the 3 incubation conditions indicated that leg morphological development was significantly advanced by light exposure during embryogenesis. Tibias from embryos incubated in 24L from E0 to E19 were 12% longer than tibias from embryos incubated in 12L for the same duration, and almost 20% longer than tibias from embryos incubated in 24D (Fig. 2, Table S1). We interpret these differences as indicators of accelerated growth, rather than enhanced or atypical growth, because we found no differences in body weight at hatching between chicks incubated in these conditions in our earlier study, despite a 1–2 day difference between groups in time to hatch [6]. Also, accelerated growth in light-exposed chick embryos has been reported in multiple studies that found normal rates of hatching, suggesting that development was not impacted negatively by the exposure [2], [3], [6], [26], [30], [31]. We too found no indicators in the current or previous study, such as deformity, abnormal growth or abnormal behavior to suggest that there was a biological cost associated with the accelerated growth.

The embryos incubated in 24L conditions produced significantly more bilateral RLMs and bilateral RLM cycles, even after normalizing for differences in experiment duration, suggesting to us that light exposure also accelerated the development of locomotor circuits. Our previous study indicated that light may advance precocious locomotor skill by as much as 2 days. However, we could not rule out that early hatching only unmasked locomotor potential established well before hatching [6]. Nonetheless, findings in an earlier study of motor activity at E18 appeared to indicate that light exposure accelerated development of respiratory motor control [5], and the array of physical, physiological and behavioral findings across our studies suggest to us that light exposure during embryogenesis accelerates development of many, if not all, body systems. For these reasons we propose that the greater incidence of bilateral RLMs is evidence that embryos incubated in 24L conditions were neurologically more mature than embryos incubated in 12L and 24D; and embryos incubated in 24D conditions were least mature. As explained in Methods, embryos incubated in 12L served as a control group for comparisons at a common incubation time point (E19), 2 days before their hatch date. We also propose that embryos incubated in 24L conditions and tested at E19, 1 day before their hatch date, were developmentally similar to E20 embryos that hatch on E21 under standard incubation conditions; and embryos incubated in 24D and tested at E19, 3 days before their hatch date, were developmentally similar to E18 embryos incubated under standard conditions. Though our study lacked control recordings at E18 or E20, our current findings for embryos incubated in 24L conditions were similar to earlier recordings at E20 from embryos incubated under standard conditions [23]. It will be important to test and validate the age equivalences in future studies. Nonetheless, we further propose that our light exposure conditions are a valuable experimental assay for examining developmental processes.

To what extent there is a critical window for inducing these developmental effects has yet to be experimentally determined, but available evidence suggests that light may have greatest effect during the first days of incubation and least effect in the final week before hatching [25]. The mechanisms underlying light-accelerated morphogenesis are not well understood, however light appears to impact cellular and molecular levels of development. Light has been shown to accelerate blastoderm expansion in chick embryos within 20 hours of exposure compared to dark-incubated controls [2]. Also, continuous light exposure during the first day of embryogenesis increases the number of differentiated somites at E2 [30]. It has been proposed that light induces accelerated growth in chicks by stimulating mitosis of neural crest ectoderm and adjacent mesoderm, hastening neural tube closure and somite formation [32]. In mammalian cellular assays, light enhances metabolism by increasing intracellular cAMP levels. For example, light wave irradiation of monolayer cultures of Chinese hamster cells elevated the levels of intracellular cAMPs [33], which are critical second messengers for DNA synthesis [34]. Evidence in chick embryos also indicates that the differentiation rate of fetal myoblasts into adult myoblast cells doubled by E15, when embryos were continuously exposed to green light (560 nm) beginning E5 [35]. Further, the rate of embryogenesis in response to light exposure has been found to differ between strains of chicks, suggesting that light exposure targets specific molecular mechanisms to accelerate morphogenesis [36].

Light exposure can also influence growth and development by modifying circadian function. The circadian system regulates physiological functions, such as heart rate and body temperature, and behaviors, including feeding and locomotion, by controlling melatonin production [37]. Melatonin influences cell proliferation and accelerates development in zebrafish embryos [38], and stimulates secretion of growth hormone in humans [39]. However, the roles of melatonin and circadian function during embryogenesis in chicks are not yet understood. The pineal gland differentiates by E3 in chick embryos [40], but melatonin release is not detected until E13–E14 [41], and photo entrainment is not established before E13 [42], [43]. Further, regulation of the circadian clock gene in the suprachiasmatic nucleus is first detected at E16, in the pineal gland at E18 [44], and in retina photoreceptors at E19 [45]. Hence, it is possible that in chick, light-induced acceleration of early embryogenesis is through mechanisms that are not dependent upon circadian mechanisms. However, circadian mechanisms positively influence postnatal growth in chicks [35], [46], and may begin to impact embryonic development several days before hatching. Light exposure can also influence the development of skeletal muscle fibers [47], and muscle activity, that in turn, may modulate growth of bones [48]. Thus, we cannot rule out that the evidence in our study of accelerated morphological and locomotor-related development might be at least partially attributed to the effects of increased muscle activity.

### Light Accelerates Development of Interlimb Coordination

Results indicated that the incidence of bilateral RLMs varied positively with light exposure. After adjusting for differences in recording time across experiments, we found that embryos incubated in continuous bright light (24L) produced more bilateral RLMs than other embryos (Table 1). Also, both the TA and LG were active bilaterally during many of the same RLM sequences (Fig. 3D, 3F, 3A). In contrast, embryos incubated in 24D conditions produced few bilateral RLMs despite producing many unilaterally, and few of the bilateral RLMs included both TA and LG burst sequences (Fig. 5C1). Light exposure also appeared to accelerate development of alternating muscle activity in the left and right legs characteristic of bipedal stepping. Embryos incubated in 24L produced many alternating left-right burst sequences during both TA and LG RLMs (Fig. 3D, 3F, 5A1), and in all cases, average relative phase fell between 0.4–0.6 (Tables S2, S4), typical of symmetric bipedal locomotion at hatching [49], [50]. Embryos incubated in 12L or 24D, on the other hand, often exhibited a broad distribution in relative phase for bilateral TA and LG RLMs (Fig. 5B, 5C, Fig. 7B1–C2), and average relative phase fell outside the 0.4–0.6 range in 43% to 67% of experiments (Tables S2, S4). Circular statistics for vector direction did not find a significant difference in relative phase; indeed there were only 4 instances where the average relative phase was less than 0.25, suggesting that there was a general bias for left-right alternation over that for synchrony (Fig. 8). Collectively, we interpret these measures as indicators that bilateral RLMs for 12L and 24D conditions were less well organized and interlimb coordination less mature. However, the greater incidence of symmetric, alternating RLMs appears to suggest that light exposure in 24L conditions accelerated development of bilateral stepping in preparation of precocious walking.

Under 24L conditions, light also appeared to preferentially enhance development of TA RLMs over that of LG RLMs. Bilateral TA RLMs were observed more frequently than LG RLMs (Fig. 4A, Table 1) and more bilateral TA cycles were typically generated within experiments than LG cycles (Fig. 8A, Table S3). The prevalence of TA RLMs suggested to us that there may be a flexor bias in RLM development. A flexor bias was previously observed in the first studies to identify and characterize RLMs at E18 and E20 [22], [23]. We interpret the greater incidence of bilateral TA RLMs in embryos incubated in 24L as further evidence of a flexor bias in recruitment of motor neurons by the spinal locomotor generator in the final phase of embryogenesis preceding precocious locomotion. The functional significance of a flexor bias in late embryogenesis is not known but may well be an evolutionary accommodation for late-stage growth in a fixed space.

### Development of Locomotor Circuitry

Given that chicks take their first steps within hours after hatching, it is likely that the neural circuitry for locomotion is assembled before hatching. EMG studies of intralimb control suggest that control of unilateral stepping gradually emerges in the last 4–5 days before hatching [22], [23]. Flexor and extensor muscles of the hip, knee and ankle are recruited during RLMs in patterns similar to those during locomotion, and muscle burst frequencies fall within the same range as kinematic and EMG burst frequencies for walking, swimming and airstepping at hatching [23], [51]. Pilot EMG recordings at E20 also suggest that left-right coordination emerges at least 1 day before hatching [23]. Our study extends these findings by providing evidence that the circuitry for interlimb stepping is established over the final 2–3 days before hatching and that emergence of bilateral RLMs follows a behavioral sequence.

The notion that limb control for locomotion is the product of a developmental sequence during embryogenesis was posed nearly 30 years ago [52], but to our knowledge the hypothesis was never experimentally tested. Based on her pioneering EMG studies in chick embryos [17], [19] and classic works by Hamburger and colleagues [18], [53], [54], Bekoff hypothesized that coordination begins first within a joint by establishing control of antagonist muscles crossing that joint. Multiple joints are then yoked for intralimb coordination and multiple limbs are yoked thereafter for interlimb coordination. The sequence appeared to establish intralimb coordination by E9 [19], [20] and available evidence suggested interlimb coordination might not emerge until the onset of hatching [52]. Our study provides experimental evidence supporting Bekoff’s hypothesis by employing light to vary the rate of maturation. Embryos incubated in 24L exhibited the most mature leg development and bilateral alternating RLM control. Whereas embryos incubated in 24D exhibited delayed morphologic development, more unilateral than bilateral RLMs, and when they produced bilateral EMG activity, interlimb coordination was less well defined. Nonetheless, RLM burst frequencies for TA or LG (Table 2) did not vary with differences in maturation or interlimb control, suggesting that the temporal structure for stepping was established prior to control of interlimb coordination. Because the purpose of our study was to determine if chicks that hatched early after 24L incubation also exhibited an earlier maturation of interlimb coordination in ovo, our experimental design only included a control for 1 time point. Thus a descriptive developmental study of interlimb coordination examining multiple time points will be essential to confirm or otherwise extend our understanding of the underlying preparations for precocious walking in the chick.

Thus, assuming the differences in maturity across the 3 incubation conditions approximated ±1 day differences in normal development, we propose that there is a sequence in the assembly of locomotor circuitry during the final 3–4 days of embryogenesis that accounts for the transformation from intralimb to interlimb RLM control and preparation for precocious locomotion. In specific, intralimb RLM control is the first to emerge, though the combination of rhythmically active muscles can vary [22], [23]; interlimb RLMs are infrequent during this phase. When bilateral RLMs first occur, the relative phase of contralateral leg muscle activity is randomly distributed, but there is a bias for interlimb alternation over that for synchrony. The distribution of relative phase narrows with maturation of bilateral RLMs and left-right alternating flexor or extensor bursting is the default interlimb pattern. Though synchronous bursting was observed during bilateral RLMs (Fig. 7C2), it was infrequent, which may indicate that synchronous activity requires sensory reinforcement or descending control to sustain it, as during hatching [55].

Our findings also offer considerations for CPG control of locomotion. Differential activation of bilateral TA and LG RLMs provide further support for previous studies arguing that CPG rhythm and pattern are separately controlled [22], [23], [56], [57]. The apparent preferential drive of bilateral TA RLMs and the activation of LG RLM independently of TA RLMs suggest that interlimb control may be somewhat modular during development. Further study of the differential control in TA and LG RLMs may also inspire further understanding of how a CPG network remains flexible in response to modulatory inputs such as limb unloading and posture. The basal ganglia have been incorporated in more recent CPG modeling and proposed to control CPG selection via inhibition [58]. Based on this model, we speculate that the differential generation of bilateral TA and LG RLMs during late stage development is related to the extreme postural flexion in ovo. The associated sensory cues may not only interact with spinal CPG circuits, but also with higher neural centers, such as the basal ganglia, to implement optional modular control for variations in leg motor patterns as during walking, airstepping, swimming and hatching.

### Significance of Findings

In this study we provide novel findings regarding the impact of light on embryogenesis. We show that light can modulate the rate of morphological development and motor control development without apparent negative consequences. These findings further suggest that light can also accelerate the neural and physiological functions required for support of precocious locomotion at hatching. The findings lead us to conclude that light exposure during early development warrants further exploration as a tool for scientific inquiry as well as for its potential economic and therapeutic benefits.

## Supporting Information

Table S1 **Tibia length at E19.** Tibia length varied significantly across the 3 incubation conditions of 24L, 12L and 24D. Tibia length was greatest for 24L conditions and least for 24D conditions.
(DOCX)

Table S2 **Summary of relative phase analyses.** The incidence of significant Rayleigh’s tests and average relative phase between 0.4–0.6 for bilateral TA RLMs varied across the 3 incubation conditions of 24L, 12L and 24D.
(DOCX)

Table S3 **Rayleigh’s circular statistics analyses for randomness.** Details of Rayleigh’s test results are summarized for both bilateral TA and LG RLM cycles for all experiments. In general, larger samples yielded relative phase distributions significantly different from random. Relative phase distributions for smaller samples were less likely to differ from random.
(DOCX)

Table S4 **Details for vectors in** **Figure 8** **.** Details for vector direction and length are provided for all.
(DOCX)
