# Supplementary material for: Light Accelerates Morphogenesis and Acquisition of Interlimb Stepping in Chick Embryos
Source: PLoS One. 2012 Dec 6;7(12):e51348. doi: 10.1371/journal.pone.0051348 (PMC3516530; doi:10.1371/journal.pone.0051348)
Supplement: Table S1 — Tibia length at E19. Tibia length varied significantly across the 3 incubation conditions of 24L, 12L and 24D. Tibia length was greatest for 24L conditions and least for 24D conditions. (DOCX) [file pone.0051348.s001.docx]

Table S1: Tibia length at E19.

| **Incubation Condition** | **Left Tibia Length (mm)^1^** |
| --- | --- |
| **24L** | 29.4 ± 1.3 |
| **12L** | 25.9 ± 0.8 |
| **24D** | 23.9 ± 1.3 |

Values represent mean ± SD
^1^Two-way ANOVA, main effect for incubation condition, p<0.001
