# Supplementary material for: Light Accelerates Morphogenesis and Acquisition of Interlimb Stepping in Chick Embryos
Source: PLoS One. 2012 Dec 6;7(12):e51348. doi: 10.1371/journal.pone.0051348 (PMC3516530; doi:10.1371/journal.pone.0051348)
Supplement: Table S2 — Summary of relative phase analyses. The incidence of significant Rayleigh’s tests and average relative phase between 0.4–0.6 for bilateral TA RLMs varied across the 3 incubation conditions of 24L, 12L and 24D. (DOCX) [file pone.0051348.s002.docx]

Table S2: Summary of relative phase analyses.

| **Incubation Condition** | **Rayleigh’s test TA^1^** | **0.4-0.6**  **TA^2^** | **Rayleigh’s test LG^1^** | **0.4-0.6**  **LG^2^** |
| --- | --- | --- | --- | --- |
| **24L** | 8 of 8 | 8 of 8 | 9 of 9 | 8 of 9 |
|  |  |  |  |  |
| **12L** | 5 of 9 | 4 of 9 | 4 of 6^3^ | 4 of 7 |
|  |  |  |  |  |
| **24D** | 4 of 7^3^ | 3 of 9 | 1 of 7^3^ | 5 of 8 |

^1^Number of experiments different from random distribution

^2^Number of experiments with average relative phase values between 0.4-0.6 (See table S3 and S4 for further details)

^3^Rayleigh test cannot be applied if sample size (n) < 6
