# Supplementary material for: Light Accelerates Morphogenesis and Acquisition of Interlimb Stepping in Chick Embryos
Source: PLoS One. 2012 Dec 6;7(12):e51348. doi: 10.1371/journal.pone.0051348 (PMC3516530; doi:10.1371/journal.pone.0051348)
Supplement: Table S3 — Rayleigh’s circular statistics analyses for randomness. Details of Rayleigh’s test results are summarized for both bilateral TA and LG RLM cycles for all experiments. In general, larger samples yielded relative phase distributions significantly different from random. Relative phase distributions for smaller samples were less likely to differ from random. (DOCX) [file pone.0051348.s003.docx]

Table S3: Rayleigh’s circular statistics analyses for randomness.

| **Experiment** | **TA Cycles (n)** | **P** | **LG Cycles (n)** | | **P** | |
| --- | --- | --- | --- | --- | --- | --- |
| **24L** |  |  |  | |  | |
| 1 | 29 | <0.001 | 30 | | <0.001 | |
| 2 | 35 | <0.001 | 35 | | <0.001 | |
| 3 | 108 | <0.001 | 33 | | <0.001 | |
| 4 | 270 | <0.001 | 21 | | <0.005 | |
| 5 | 745 | <0.001 | 27 | | <0.001 | |
| 6 | 122 | <0.001 | 260 | | <0.001 | |
| 7 | 157 | <0.001 | 116 | | <0.001 | |
| 8 | 43 | <0.001 | 40 | | <0.001 | |
| 9 |  |  | 52 | | <0.001 | |
|  |  |  |  | |  | |
| **12L** |  |  |  | |  | |
| 1 | 22 | <0.003 | 25 | | <0.004 | |
| 2 | 18 | >0.05 | 4 | | NA* | |
| 3 | 39 | >0.6 | 37 | | <0.005 | |
| 4 | 13 | >0.4 | 25 | | <0.001 | |
| 5 | 8 | <0.001 | 15 | | >0.26 | |
| 6 | 16 | <0.001 | 59 | | <0.001 | |
| 7 | 66 | <0.001 | 6 | | >0.3 | |
| 8 | 6 | >0.1 |  | |  | |
| 9 | 13 | <0.02 |  | |  | |
|  |  |  |  | |  | |
|  |  |  |  | |  | |
| **24D** |  |  |  | |  | |
| 1 | 3 | NA* | 3 | | NA* | |
| 2 | 16 | >0.1 | 13 | | >0.6 | |
| 3 | 10 | <0.03 | 28 | | <0.001 | |
| 4 | 18 | >0.1 | 8 | | >0.3 | |
| 5 | 3 | NA* | 19 | | >0.2 | |
| 6 | 7 | <0.002 | 10 | | >0.1 | |
| 7 | 9 | <0.03 | 32 | | >0.05 | |
| 8 | 22 | <0.001 | 21 | | >0.2 | |
| 9 | 22 | >0.3 |  | |  | |
|  |  |  |  |  | |  |

*Note: Rayleigh test cannot be applied if sample size (n) < 6
