# Supplementary material for: Light Accelerates Morphogenesis and Acquisition of Interlimb Stepping in Chick Embryos
Source: PLoS One. 2012 Dec 6;7(12):e51348. doi: 10.1371/journal.pone.0051348 (PMC3516530; doi:10.1371/journal.pone.0051348)
Supplement: Table S4 — Details for vectors in Figure 8 . Details for vector direction and length are provided for all. (DOCX) [file pone.0051348.s004.docx]

Table S4: Details for vectors in Figure 8.

| **Experiment** | **TA Vector** | **TA Vector** | **LG Vector** | **LG Vector** |
| --- | --- | --- | --- | --- |
|  | direction | length* | direction | length* |
| **24L** |  |  |  |  |
| 1 | 0.54 | 6 | 0.47 | 3 |
| 2 | 0.44 | 6 | 0.48 | 7 |
| 3 | 0.50 | 14 | 0.46 | 6 |
| 4 | 0.40 | 90 | 0.43 | 3 |
| 5 | 0.45 | 124 | 0.46 | 9 |
| 6 | 0.44 | 29 | 0.49 | 43 |
| 7 | 0.46 | 40 | 0.41 | 40 |
| 8 | 0.42 | 10 | 0.49 | 10 |
|  |  |  | 0.38 | 12 |
|  |  |  |  |  |
| **12L** |  |  |  |  |
| 1 | 0.24 | 6 | 0.41 | 7 |
| 2 | 0.31 | 14 | 0.20 | 3 |
| 3 | 0.41 | 13 | 0.35 | 7 |
| 4 | 0.43 | 3 | 0.47 | 5 |
| 5 | 0.43 | 2 | 0.35 | 4 |
| 6 | 0.19 | 5 | 0.47 | 21 |
| 7 | 0.38 | 23 | 0.45 | 1 |
| 8 | 0.43 | 2 |  |  |
| 9 | 0.31 | 2 |  |  |
|  |  |  |  |  |
| **24D** |  |  |  |  |
| 1 | 0.34 | 1 | 0.38 | 1 |
| 2 | 0.35 | 3 | 0.43 | 3 |
| 3 | 0.33 | 3 | 0.27 | 5 |
| 4 | 0.41 | 5 | 0.41 | 2 |
| 5 | 0.51 | 1 | 0.46 | 7 |
| 6 | 0.19 | 6 | 0.47 | 9 |
| 7 | 0.28 | 2 | 0.40 | 9 |
| 8  9 | 0.45  0.39 | 6  5 | 0.31 | 4 |

* Vector length normalized to number of reference cycles/hr
